# Dysregulated autophagy at the nexus of type 2 diabetes mellitus and pancreatic cancer pathogenesis

**DOI:** 10.3389/fendo.2025.1735726

**Published:** 2026-01-12

**Authors:** Longmei Li, Yatong Chen, Shushu Wang, Wanying Du, Tianfeng Lao, Feng Chen, Gengzhen Yao, Wensheng Chen, Xuewen Li, Chunping Liu, Xiaoguang Xue

**Affiliations:** 1 State Key Laboratory of Traditional Chinese Medicine Syndrome, The Second Affiliated Hospital of Guangzhou University of Chinese Medicine, Guangzhou, China; 2 Chinese Medicine Guangdong Laboratory, Guangzhou University of Chinese Medicine, Zhuhai, Guangdong, China; 3 College of Basic Medicine, Guangzhou University of Chinese Medicine, Guangzhou, Guangdong, China; 4 School of Biology and Food Engineering, Changshu Institute of Technology, Changshu, Jiangsu, China

**Keywords:** autophagy, comorbidity, diabetes mellitus, molecular mechanism, pancreatic cancer

## Abstract

Diabetes Mellitus (DM) and Pancreatic Cancer (PC) are two major diseases that pose severe threats to global health, and their comorbidities have drawn increasing attention in recent years. Substantial epidemiological evidence indicates that type 2 diabetes mellitus (T2DM) is not only a risk factor for PC development but also an early clinical manifestation. The pathogenesis of these two conditions is interactive, wherein autophagy—a highly conserved intracellular catabolic process—plays a central role in maintaining pancreatic β-cell function, regulating insulin sensitivity, and determining the fate of PC cells. In diabetes, dysregulated autophagy contributes to β-cell failure and insulin resistance; in PC, autophagy plays a dual role, potentially suppressing tumorigenesis in early stages while supporting cancer cell survival and proliferation during progression, thereby mediating chemotherapy resistance. We review the epidemiological links, shared risk factors, and mutual influences of clinical management strategies in the comorbidity of diabetes and PC and focus on dissecting the pivotal role of autophagy at the intersection of their mechanisms. To elucidate the connection of autophagy pathways in both diseases, we aimed to provide novel perspectives on their common pathophysiological basis and identify potential targets for innovative therapeutic strategies against this comorbidity.

## 1 Background

Diabetes mellitus is a global metabolic disorder characterized by chronic hyperglycemia, primarily arising from insulin secretion deficiency due to pancreatic β-cell dysfunction and insulin resistance in peripheral tissues (1). According to the latest data from the International Diabetes Federation (IDF), the global diabetic population exceeds 537 million and is projected to rise to 783 million by 2045, with type 2 diabetes mellitus (T2DM) accounting for over 90% of cases (2). Pancreatic cancer, particularly pancreatic ductal adenocarcinoma (PDAC), ranks among the most lethal digestive tract malignancies, with a five-year survival rate of approximately 10% (3). It is projected to become the second leading cause of cancer-related deaths by 2030 (4). Pancreatic cancer onset is insidious, early diagnosis is challenging, and most patients are diagnosed at advanced stages, missing the window for curative surgery.

Clinical observations have long noted a close association between diabetes and pancreatic cancer. This relationship is bidirectional: on one hand, long-term T2DM (>5 years) is a well-established risk factor for pancreatic cancer, increasing the risk by 1.5 to 2.0-fold, and up to 50%-80% of pancreatic cancer patients present with hyperglycemia or new-onset diabetes at diagnosis, the latter often considered a paraneoplastic syndrome or early sign of pancreatic cancer (5–7). This comorbidity suggests shared upstream pathogenic mechanisms. Traditionally, chronic inflammation, hyperinsulinemia, and activation of the insulin-like growth factor-1 (IGF-1) signaling pathway have been considered to link diabetes and pancreatic cancer (8–12). In recent years, the crucial role of autophagy, a fundamental cellular homeostatic regulatory mechanism, has been increasingly revealed.

Autophagy is an evolutionarily conserved process in eukaryotic cells that involves the degradation of damaged organelles, misfolded proteins, and pathogens via lysosomes (13, 14). The mode of substrate delivery to lysosomes can be categorized into macroautophagy, microautophagy, and chaperone-mediated autophagy, with the term “autophagy” commonly referred to as macroautophagy (15). Basal autophagy is essential for maintaining cellular health, particularly in metabolically active and stress-prone cells such as pancreatic β-cells. In diabetes, dysfunctional autophagy leads to the accumulation of damaged mitochondria and endoplasmic reticulum (ER) in β-cells, triggering oxidative stress and ER stress and ultimately promoting β-cell apoptosis and insulin secretion defects (16, 17). An imbalance in insulin of autophagy target tissues also exacerbates insulin resistance (18). In pancreatic cancer, the role of autophagy is more complex and context-dependent. During the precancerous lesion stage, autophagy acts as a tumor suppressor by clearing damaged components and suppressing inflammation; however, in established pancreatic tumors, cancer cells harness autophagy to cope with metabolic stresses such as nutrient deprivation, hypoxia, and chemotherapy, whereby autophagy activation supports tumor proliferation, invasion, metastasis, and drug resistance (19–21). We propose that, autophagy emerges is a key molecule connecting the metabolic dysregulation of diabetes with the malignant biology of pancreatic cancer. In-depth exploration of the dynamic changes and regulatory networks associated with comorbidities has significant theoretical and clinical implications for disease prevention, early diagnosis, and combination therapy.

## 2 Bidirectional associations between diabetes duration, glycemic control, and pancreatic cancer risk

The epidemiological association between diabetes and pancreatic cancer has been confirmed by numerous prospective cohort studies, case-control studies, and meta-analyses. A large cohort study involving over 1 million Asian individuals reported a summary relative risk (RR) of pancreatic cancer of 1.76 among diabetic patients, with the risk increasing with the duration of diabetes (22). The association between new-onset diabetes (within 3 years of diagnosis) and pancreatic cancer risk is particularly strong, with RRs as high as 2.0 to 2.5, strongly suggesting that new-onset diabetes may be an early clinical manifestation of pancreatic cancer (23). It is notable that the endocrine and exocrine pancreases share a common vascular bed. Through this shared vasculature, soluble factors can mediate pathogenic cross-talk between the two compartments (24). For example, TGF-β signaling upregulated during PDAC progression may traverse this network and contribute to β-cell exhaustion (25).

In addition to duration, the treatment modality for diabetes is also associated with pancreatic cancer risk, providing indirect evidence for a causal relationship. Multiple retrospective studies have shown that long-term metformin use is associated with a reduced risk of pancreatic cancer, potentially mediated by metformin’s activation of AMP-activated protein kinase (AMPK) and inhibition of mammalian target of rapamycin (mTOR) signaling by metformin, thereby modulating autophagy (26–28). Conversely, some studies suggest that long-term use of exogenous insulin or insulin secretagogues may slightly increase pancreatic cancer risk, possibly because hyperinsulinemia persistently activates the insulin/IGF-1 receptor pathway and promotes cell proliferation (28, 29). However, whether these associations are subject to bias and their definitive causality require further prospective validation.

From the perspective of pancreatic cancer, diabetic patients constitute a significant proportion of pancreatic cancer patients. Pooled analyses indicate that approximately 25%-50% of pancreatic cancer patients have comorbid diabetes, with approximately 40%-50% being newly diagnosed within the preceding three years (6, 30–32). Pancreatic cancer patients with comorbid diabetes often have a poorer prognosis and shorter overall survival (33). This may be attributed to multiple factors, including a hyperglycemic environment promoting tumor progression, diabetic microvascular complications limiting the full application of chemotherapy, and generally poorer overall health status in the comorbid state (34–36). Furthermore, genetic epidemiological studies have revealed that certain polymorphisms associated with diabetes and insulin resistance are also linked to pancreatic cancer susceptibility, supporting the notion that these two diseases share part of their genetic background (37, 38).

In summary, robust epidemiological evidence has established a close association between diabetes (particularly new-onset diabetes) and pancreatic cancer. This relationship manifests not only in diabetes being a risk factor for pancreatic cancer but also in pancreatic cancer-induced diabetes. This bidirectional interplay points to an interaction in their pathophysiological mechanisms, we propose that autophagy is a common pathway that likely plays a critical role.

## 3 Role of autophagy dysfunction in linking diabetes to pancreatic cancer pathogenesis

**Figure 1:**
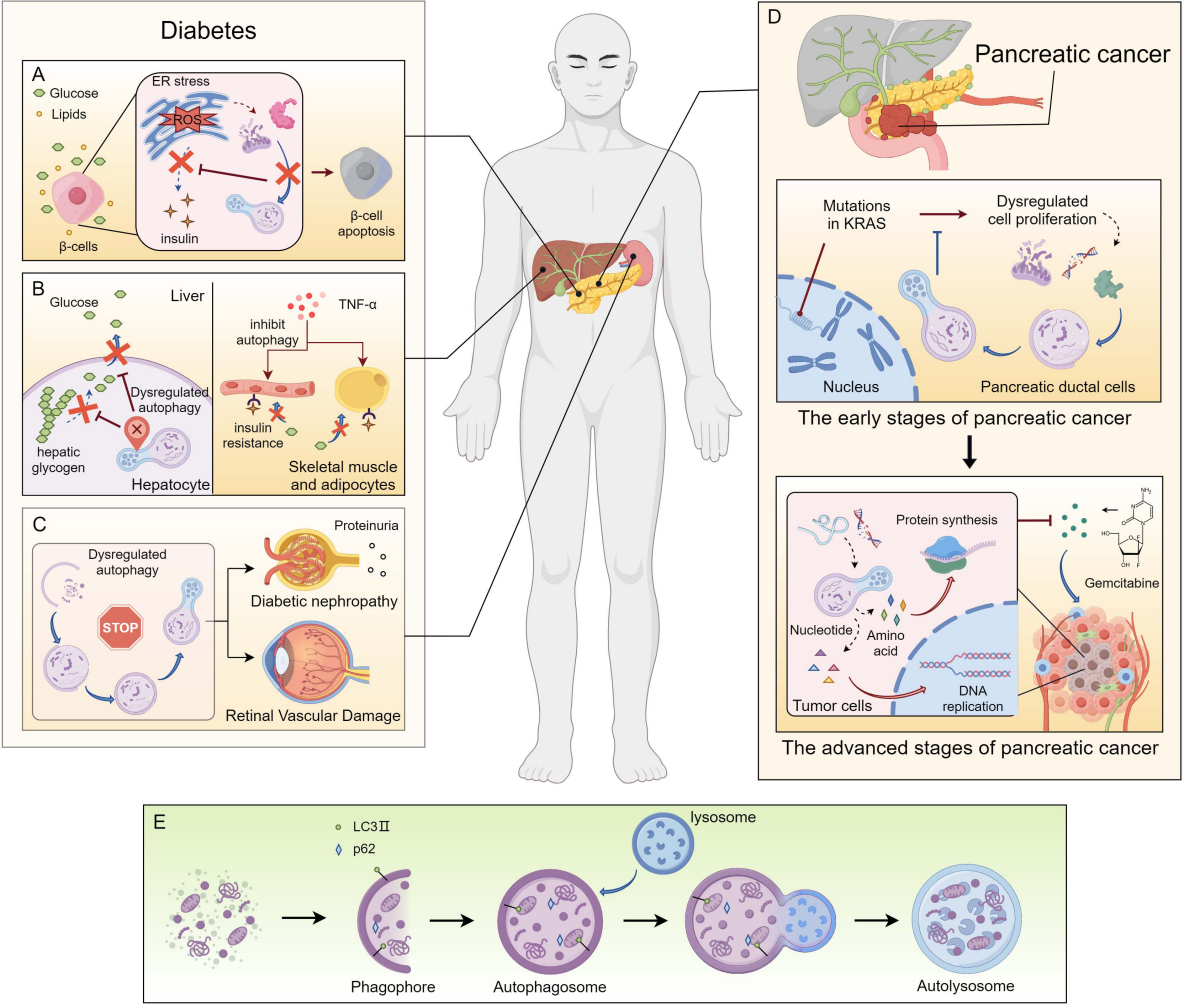
The Dual Role of Autophagy in Linking Type 2 Diabetes and Pancreatic Cancer Pathogenesis. **(A)** Impaired autophagy leads to disruption of β-cell homeostasis. **(B)** Dysregulated autophagy causes glucose metabolic dysregulation in insulin target tissues. **(C)** Autophagic dysfunction drives diabetic complications. **(D)** Dual role of autophagy in pancreatic tumorigenesis and progression. **(E)** Core process of autophagy.

As a core homeostatic regulatory mechanism, autophagy plays an indispensable role in maintaining normal pancreatic physiology. The pancreas, which performs both exocrine (digestive enzyme secretion) and endocrine (insulin, glucagon secretion) functions, comprises highly metabolically active and stress-prone cell types—acinar cells and pancreatic β-cells—that critically rely on autophagy to clear damaged components and maintain function (39, 40). In diabetes, disrupted autophagy is involved in the core pathologies of β-cell failure and insulin resistance (18, 41); in pancreatic cancer, autophagy involves a complex biological mechanisms, acting as a potential tumor suppressor mechanism and a survival dependency for established tumors (42). It is worth noting that the dysregulation of autophagy is increasingly regarded as a key mechanism connecting the risk factors for diabetes and pancreatic cancer. In this section, we systematically explore the specific role of autophagy in the pathogenesis of diabetes and pancreatic cancer, and elucidate its function as a molecular connection linking these two diseases in their comorbid context (**Figure 1**).

### 3.1 Impaired autophagy as a pathogenic driver of diabetes

The role of autophagy in diabetes is tissue and cell-specific, with its function in pancreatic β-cells being the most extensively studied. β-cells are responsible for sensing blood glucose levels and precisely secreting insulin; their functional integrity is crucial for maintaining glucose homeostasis (43). Basal autophagy is essential for β-cell survival and function. Mice with β-cell-specific knockout of the essential autophagy gene Atg7 exhibit reduced β-cell mass, abnormal insulin secretory granules, defective insulin secretion, and ultimately develop overt diabetes (44). Evidence of impaired autophagic flux, including accumulated autophagosomes and reduced lysosomal function, has also been observed in islets from human T2DM patients (45). Under metabolic stress from high glucose and lipids (glucolipotoxicity), ER stress and oxidative stress intensify within β-cells, generating abundant misfolded proteins and damaged mitochondria (46). A fully functional autophagic system is crucial under these conditions to clear these harmful substances and maintain cell survival. If autophagy is deficient, stress signals persist, eventually triggering β-cell apoptosis.

In addition to β-cells, autophagy plays key roles in insulin-target tissues. In the liver, autophagy participates in regulating glycogen metabolism and gluconeogenesis. During starvation, autophagy is activated, degrading intracellular components to produce precursors like amino acids for gluconeogenesis, thereby maintaining blood glucose (47). In insulin-resistant states, hepatic autophagy may become dysregulated, contributing to abnormal hepatic glucose output (48). In skeletal muscle and adipose tissue, autophagy also participates in metabolic regulation. Research indicates that autophagy function is altered in skeletal muscle and adipocytes under conditions of obesity and insulin resistance, potentially through the activation of inflammatory signaling pathways (49, 50). For example, inflammatory cytokines like tumor necrosis factor-s (TNF-α) can inhibit autophagy, and autophagy inhibition can further exacerbate inflammation, creating a vicious cycle that exacerbates insulin sensitivity (51).

Autophagy is also closely involved in the development and progression of diabetic complications. In diabetic nephropathy, impaired autophagy in glomerular podocytes in high-glucose environments leads to cell injury and proteinuria, accelerating nephropathy progression (52). In diabetic retinopathy, dysregulated autophagy in retinal pericytes similarly contributes to vascular damage (53). Therefore, modulating autophagy has emerged as a potential novel strategy for treating diabetes and its complications. For example, some of the beneficial effects of metformin are attributed to its activation of AMPK, which thereby mildly enhances autophagy (54). Rapamycin (an mTOR inhibitor) is a potent autophagy inducer, but its systemic side effects limit clinical application (55). Identifying more precise means of autophagy modulating is a research direction.

This section details the multifaceted roles of autophagy in diabetes. From preserving βreser function to modulating metabolism in insulin-targeted tissues and contributing to various complications, autophagy is critically involved at multiple pathophysiological levels. This widespread involvement underscores that autophagy dysfunction is a common thread linking diverse diabetic pathologies.

### 3.2 Dual role of autophagy in pancreatic tumorigenesis and progression

In pancreatic cancer, autophagy plays a seemingly paradoxical role, and its function is highly dependent on the stage of tumor development and the microenvironment.

In the early stages of pancreatic cancer development, autophagy primarily acts as a tumor suppressor. Mutations in the oncogene Kirsten rat sarcoma virus (KRAS) are the most common and critical initiating event in pancreatic cancer and are found in more than 90% of PDAC cases (56). At the precancerous lesion stage, activated KRAS initially promotes autophagy, likely as a cell-autonomous adaptation mechanism to clear metabolic waste and damaged organelles generated by KRAS-driven proliferation, thereby maintaining cellular homeostasis and preventing genomic instability. Inhibiting autophagy at this stage accelerates KRAS-driven pancreatic intraepithelial neoplasia (PanIN) progression to invasive carcinoma (57). Furthermore, deletion of autophagy-related genes has been shown to promote pancreatic inflammation and fibrosis, creating a microenvironment favorable for tumorigenesis (57, 58).

However, once pancreatic cancer is fully established and progresses to advanced stages, the role of autophagy shifts from beneficial to harmful. Advanced pancreatic cancer is characterized defined by an extremely desolate tumor microenvironment: dense fibrotic stroma and poor vascular supply lead to severe nutrient and oxygen deprivation (59). Under these extreme stress conditions, the survival of cancer cells relies heavily on autophagy. Autophagy sustains cancer cells by degrading intracellular macromolecules, providing basic raw materials necessary for maintaining essential metabolism, biosynthesis, and energy production, thereby supporting their survival and growth in harsh environments (60). Numerous preclinical studies have confirmed that inhibiting autophagy, either genetically or pharmacologically, in established pancreatic cancer models, suppresses tumor growth and synergizes with standard chemotherapies such as gemcitabine, enhancing anti-tumor effects (61–63).

Autophagy is also a key mechanism of chemotherapy resistance in pancreatic cancer. Chemotherapeutic agents themselves represent strong stressors to cancer cells and can induce autophagy. Cancer cells exploit this induced autophagy to “digest” the damage inflicted by the drugs, evading apoptosis and thus developing resistance (64). Consequently, the combination of autophagy inhibitors is considered a promising strategy to overcome chemotherapy resistance in pancreatic cancer. Currently, several clinical trials are evaluating hydroxychloroquine combined with chemotherapy in pancreatic cancer, but the results suggest that targeting autophagy alone may be insufficient for significant efficacy, necessitating the use of more specific inhibitors or rational combination regimens in the future (65, 66). Moreover, to avoid off-target damage to islet β-cells from autophagy inhibition, two critical strategies can be pursued. First, autophagy modulators that specifically target the tumor microenvironment should be developed. Second, refined patient stratification in clinical practice based on islet functional reserve and tumor autophagy dependence should be implemented.

Pancreatic stellate cells (PSCs) are the primary source of the fibrotic microenvironment in pancreatic cancer (67). Recent research has revealed that PSCs in the microenvironment of pancreatic cancer are predominantly in an activated state, and their autophagy levels are also significantly increased. These findings indicate that tumor-stromal interactions can effectively activate autophagy in PSCs, thereby accelerating pancreatic fibrosis and further promoting the development and metastasis of pancreatic cancer. Inhibiting autophagy in PSCs may help remodel the tumor microenvironment, enhancing drug delivery and efficacy (68). This finding reveals that in addition to targeting tumor cells themselves, modulating autophagy in non-tumor cells within the tumor microenvironment is also a potential therapeutic avenue.

In summary, the role of autophagy in pancreatic cancer evolves from tumor suppression to tumor promotion with disease progression. Initially protective, it becomes a key survival mechanism in advanced stages, supporting cancer cells through catabolism and mediating therapy resistance. Thus, understanding this dynamic regulation is crucial for developing stage-specific therapeutic strategies.

### 3.3 Autophagy dysregulation in comorbidity pathogenesis

The comorbidity of diabetes and pancreatic cancer arises from interactions of multiple pathophysiological bases, involving a complex network of genetic susceptibility, metabolic dysregulation, and environmental exposures. Recent research suggests that dysregulation of the autophagic process may be the primary cause of these risk factors for the development of both diseases. A deep understanding of this mechanism is crucial for identifying high-risk populations and enabling early intervention.

Age is the most prominent risk factor for comorbidities, with the incidence risk steadily increasing with age, peaking particularly in individuals over 65 years of age (69). Sex differences are also evident, with males exhibiting greater susceptibility to both pancreatic cancer and T2DM (70). Studies indicate that an age-related decline in autophagic function may exacerbate metabolic stress and genomic instability in pancreatic tissue, thereby creating a common ground for the onset of both diseases (41).

Obesity, particularly central obesity, is the central metabolic connecting the two diseases, promoting their development through multiple mechanisms. Visceral adipose tissue overrelease free fatty acids, proinflammatory cytokines (including TNF-α and IL-6), and dysregulated adipokines (manifested as increased leptin and decreased adiponectin levels), collectively establishing a state of chronic low-grade inflammation (71–73). This metabolic inflammatory environment not only promotes diabetes progression by inducing insulin resistance and β-cell dysfunction but also fosters a protumor microenvironment by activating key signaling pathways such as nuclear factor kappa-B (NF-κB) and signal transducer and activator of transcription 3 (STAT3) (72, 74–76). Obesity-related metabolic pressure significantly alters autophagic activity—on the one hand, nutrient excess may suppress basal autophagy in insulin-sensitive tissues, exacerbating metabolic disorders; on the other hand, tumor cells may exploit autophagy to cope with nutrient scarcity and oxidative stress, increasing survival (60, 77).

Dietary and lifestyle factors influence disease risk through direct and indirect pathways. Long-term adherence to a Western diet pattern, high in fat, sugar, red meat, and processed meats, not only promotes obesity and metabolic abnormalities but can also alter the gut microbiota composition and increase intestinal permeability, leading to endotoxin entry into the bloodstream and further amplifying systemic inflammation (78, 79). This chronic inflammatory state interferes with the normal regulation of autophagy, placing pancreatic cells under persistent metabolic stress. Smoking, the most well-defined environmental risk factor for pancreatic cancer, involves carcinogens that directly cause DNA damage and disrupt the functional integrity of the autophagy-lysosome pathway, leading to abnormal protein aggregation and mitochondrial dysfunction (80). Alcohol intake primarily acts by inducing chronic pancreatitis, a well-established risk factor, while dysregulated autophagy during the continuous cycle of pancreatic injury and repair both accelerates endocrine failure leading to diabetes and creates favorable conditions for tumorigenesis (80).

With respect to genetic background, genome-wide association studies (GWASs) have identified several loci associated with the risk of both diseases. Transcription factor 7-like 2 (TCF7L2), one of the strongest genetic determinants of T2DM, has specific variants that are also associated with an increased risk of pancreatic cancer (81). Notably, conflicting evidence from in vitro experiments has shown that this gene plays a dual role in maintaining glucose homeostasis and regulating pancreatic epithelial cell proliferation and differentiation by modulating the Wnt signaling pathway and islet β-cell function (82, 83). Studies have shown that, TCF7L2 also participates in the regulation of autophagy, which may be a potential mechanism underlying its influence on the risk of both diseases (84). Furthermore, carriers of pancreatic cancer susceptibility genes such as BRCA1/2, PALB2, CDKN2A, and ATM not only have increased tumor risk but also the potential functions of these genes in metabolic pathways and autophagy regulation warrant further exploration (85).

Chronic pancreatitis serves is a critical connection between these two diseases. Persistent inflammatory damage and tissue repair processes lead to pancreatic fibrosis and acinar cell loss (86). In this pathological process, autophagy plays a complex dual role—moderate autophagy activation helps cells cope with metabolic stress, whereas sustained dysregulation of autophagy accelerates the destruction of the pancreatic parenchyma (87). Research indicates that autophagy abnormalities in chronic pancreatitis may simultaneously promote endocrine failure and tumorigenesis, acting as a significant driving factor of comorbidity (88, 89).

In summary, age, sex, obesity, poor diet, smoking, alcohol consumption, genetic background, and chronic pancreatitis collectively constitute the risk network for diabetes-pancreatic cancer comorbidity. These factors, by driving common downstream pathways including chronic inflammation, oxidative stress, metabolic disorders, and autophagic imbalance, form the pathological basis promoting the development and progression of both diseases. Notably, the bidirectional blood flow between the pancreatic acini and islets also provides an anatomical basis for these factors to simultaneously affect tumor cells and β cells (24). Among these, we argue that autophagy is the core mechanism for cellular quality control and metabolic adaptation, whose functional disruption may be the pathway connecting various risk factors to disease onset, offering new targets and insights for the prevention and early intervention of comorbidities.

## 4 Integrated therapeutic strategies for diabetes and pancreatic cancer comorbidity

The clinical management of diabetes and pancreatic cancer comorbidity presents a complex challenge, requiring a multidisciplinary team approach involving oncology, endocrinology, nutrition, and surgery to balance glycemic control with the efficacy and safety of antitumor therapy. Treatment strategies must be highly individualized, comprehensively considering pancreatic cancer stage, resectability, diabetes type and duration, existing complications, and the patient’s overall condition (**Table 1**).

**Table 1:** Drug therapy in the comorbidities of diabetes and pancreatic cancer.

Drug	Mechanism	Clinical application	Outcomes	Ref
Metformin	Inhibit gluconeogenesis, activate autophagy	T2DM and pancreatic cancer	Lower blood glucose, inhibit tumor cell proliferation	(27, 54, 94)
Insulin	Stimulates Glucose Uptake, inhibit hepatic glycogenolysis	T2DM	Lower blood glucose	(28)
Gemcitabine	Inhibits DNA replication	Pancreatic cancer	Inhibit tumor cell proliferation	(92, 93)
FOLFIRINOX	Induces DNA crosslinking, causes DNA Strand breaks, inhibits DNA/RNA synthesis	Pancreatic cancer	Inhibit tumor cell proliferation	(92)
Nab-paclitaxel	Blocks mitosis	Pancreatic cancer	Inhibit tumor cell proliferation	(92)
GLP-1 receptor agonists (e.g., Lixisenatide, Liraglutide, Semaglutide, Exenatide)	Inhibit hepatic glycogenolysis, regulate autophagy^*1^	T2DM	Lower blood glucose	(96, 98, 99, 101, 102)
DPP-4 inhibitor(e.g., Saxagliptin)	Inhibit hepatic glycogenolysis, activate autophagy^*2^	T2DM	Lower blood glucose	(97, 100),
SGLT2 inhibitor	Inhibit renal glucose reabsorption	T2DM	Lower blood glucose	(103)
HCQ	Inhibit autophagy	Pancreatic cancer	Enhances the cytotoxic effect of chemotherapeutic drugs on tumors	(66)

^*1^Current studies indicate that GLP-1 activators may inhibit the autophagy pathway in certain nonpancreatic tissues (e.g., the brain and ovaries). However, in β-cells, they exert protective effects by enhancing autophagy.

^*2^Recent evidence indicates that DPP-4 inhibitors can activate the autophagy pathway in tissues such as the gastric mucosa. However, their direct effects on pancreatic cells remain to be fully elucidated and require further investigation.

T2DM, type 2 diabetes mellitus; GLP-1, glucagon-like peptide-1; DPP-4, dipeptidyl peptidase 4; SGLT2, sodium-glucose transporter 2; HCQ, hydroxychloroquine.

Surgical resection remains the only potentially curative treatment for pancreatic cancer. For patients with resectable or borderline resectable pancreatic cancer comorbid with diabetes, perioperative glycemic management is paramount. Strict target glycemic control (7.8-10.0 mmol/L) helps reduce the risk of complications such as surgical site infections and anastomotic leaks (90). Pancreatic cancer surgery itself can further impair pancreatic endocrine and exocrine function, inducing or worsening diabetes, i.e., pancreatogenic diabetes (91). Therefore, close postoperative blood glucose monitoring and timely adjustment of hypoglycemic regimens are essential.

For patients with advanced pancreatic cancer, chemotherapy remains the cornerstone of treatment. Clinical cohort studies have demonstrated that common regimens such as gemcitabine, FOLFIRINOX, and nab-paclitaxel combinations, while targeting cancer cells, also impact the autophagic process through various pathways (92, 93). Gastrointestinal side effects induced by these chemotherapeutic agents can lead to reduced nutritional intake, not only increasing the risk of hypoglycemia but also altering cellular metabolic states and affecting autophagic flux (92). Consequently, during chemotherapy, alongside adjusting hypoglycemic regimens to mitigate hypoglycemia risk, attention must be given to the comprehensive impact of these drugs on autophagy pathways.

In the selection of hypoglycemic agents, careful consideration should be given to their modulatory effects on autophagy and potential synergy with antitumor therapies. Metformin, as the first-line treatment for T2DM, exerts its antitumor effects closely related to autophagy regulation—by activating AMPK and inhibiting mTOR signaling, it induces protective autophagy and suppresses tumor cell proliferation (27, 54, 94). Multiple retrospective studies indicate a survival benefit for diabetic patients with pancreatic cancer using metformin, potentially partly attributable to its enhancement of chemotherapy sensitivity via autophagic mechanisms (28, 95). Controversies regarding the pancreatic safety of glucagon-like peptide-1 (GLP-1) receptor agonists and dipeptidyl peptidase 4 (DPP-4) inhibitors have been largely addressed by large cardiovascular outcome trials, and their modulatory effects on autophagy are emerging as a new research focus (96–102). Sodium-glucose transporter 2 (SGLT2) inhibitors, while having a low risk of hypoglycemia, warrant vigilance for potential ketoacidosis, especially during reduced oral intake due to chemotherapy, as this metabolic state significantly influences autophagy levels (103). Insulin therapy should adhere to the principles of precision and moderation, avoiding potential tumor-promoting risks associated with hyperinsulinemia, while also noting the inhibitory effects of insulin on cellular autophagy (8, 104, 105).

Targeting autophagy represents a new frontier in pancreatic cancer research. Autophagy inhibitors such as hydroxychloroquine (HCQ) have been combined with chemotherapeutic agents such as gemcitabine in clinical trials for advanced pancreatic cancer (65). The rationale is to prevent tumor cells from utilizing autophagy to resist chemotherapeutic stress, thereby “starving” cancer cells. However, early clinical trial outcomes have been mixed. Several plausible explanations include: insufficient autophagy suppression in patient tumors by HCQ, the dual role of autophagy inhibition in suppressing tumor metabolism and proliferation while potentially promoting metastasis, and the current lack of suitable pharmacodynamic biomarkers (66, 106, 107). These directions may involve the development of more specific autophagy inhibitors or the exploration of combinations of autophagy modulators with immunotherapy and targeted therapy. For patients with diabetes comorbid with pancreatic cancer, coordinating the interaction between hypoglycemic drugs (e.g., metformin, an autophagy inducer) and autophagy inhibitors (e.g., HCQ) presents a novel clinical challenge (108). In summary, we argue that managing diabetes-pancreatic cancer comorbidity requires an integrated, dynamically adjusted strategy that controls metabolic disturbances while maximizing antitumor efficacy and improving patient quality of life.

## 5 Summary

The comorbidity of diabetes and pancreatic cancer represents a significant global health challenge driven by interconnected pathophysiological mechanisms. We review epidemiological, clinical, and risk factor evidence, highlighting autophagy as a central biological pathway linking these conditions.

Epidemiological studies establish diabetes—particularly new-onset cases—as both a risk factor and potential early indicator of pancreatic cancer, while pancreatic cancer frequently induces diabetes (5). This bidirectional relationship necessitates clinical vigilance in both directions. Therapeutically, management requires integrated strategies where antidiabetic treatments must be evaluated for their impact on tumor outcomes and anticancer therapies for their metabolic consequences.

Mechanistically, autophagy serves as a critical nexus. In diabetes, its impairment contributes to β-cell dysfunction and insulin resistance (40); in pancreatic cancer, it plays a context-dependent role in tumor suppression and promotion (42). Shared risk factors including obesity and chronic inflammation converge through autophagy dysregulation, creating a permissive environment for tumor development when combined with oncogenic mutations such as KRAS (56).

Key unanswered questions remain regarding the temporal dynamics of autophagy during disease progression, reliable biomarkers for risk stratification, and context-specific therapeutic modulation of autophagic pathways. We propose that addressing these issues through mechanistic studies and innovative trial designs will be essential for developing effective prevention, diagnostic, and therapeutic strategies targeting this lethal comorbidity.
